# Supplementary material for: Serotonergic neuronal death and concomitant serotonin deficiency curb copulation ability of Drosophila platonic mutants
Source: Nat Commun. 2016 Dec 13;7:13792. doi: 10.1038/ncomms13792 (PMC5159827; doi:10.1038/ncomms13792)
Supplement: Supplementary Information — Supplementary Figures 1-12 and Supplementary Note 1. [file ncomms13792-s1.pdf]

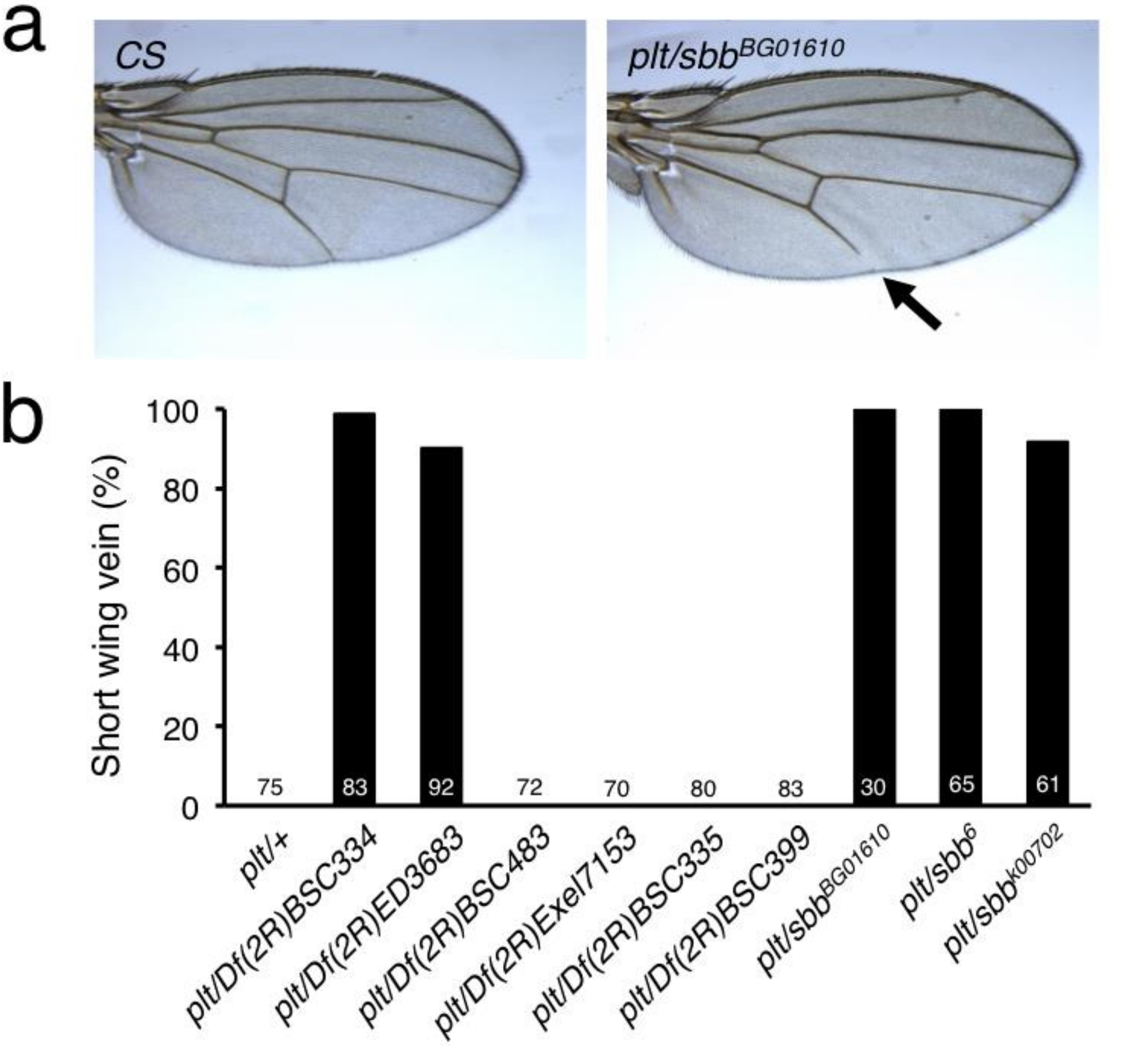

Supplementary Figure 1. The genetic complementation test of *plt* for the short wing vein phenotype (a) A wing of the *plt* mutant and wild type. **(b)** The frequencies at which flies showed the short wing vein phenotype in the genetic complementation tests. The short wing vein phenotype was not observed in *plt* heterozygotes with the wild-type (+), *Df(2R)BSC483*, *Df(2R)Exel7153*, *Df(2R)BSC335* or *Df(2R)BSC399* chromosome placed *in trans* to the *plt*-harboring chromosome. The short wing vein phenotype was observed in almost all *plt* heterozygotes with *Df(2R)BSC334*, *Df(2R)ED3683*, $sbb^{BG01610}$, sbb^{6} or sbb^{k00702} chromosome placed *in trans* to the *plt*-harboring chromosome.

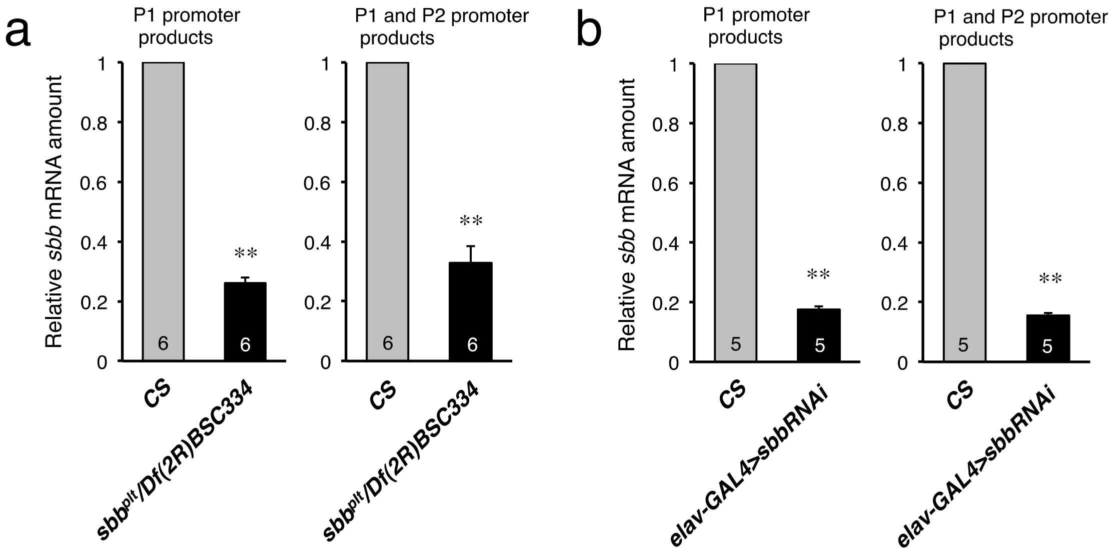

Supplementary Figure 2. Reduced *sbb* expression in *plt* mutants and the flies with *sbb* was knockdown. (**a**) RT-RCR for *sbb* transcripts in wild-type (*CS*) and *plt* mutant (*sbb*^{plt}*/Df(2R)BSC334*) flies. The *sbb* gene has two promoters, P1 and P2, and the abundance of transcripts from P1 alone and those from both P1 and P2 was quantified. (**b**) A similar analysis for flies in which *sbb* was knocked down with *sbbRNAi* in neurons (as driven by *elav-GAL4*). The genotype of flies examined was *w elav*^{C155}*-GAL4; UAS-sbb RNAi/+; UAS-Dcr2/+*. The mean ± SEM and the number of test are shown. **P<0.01 by the Mann-Whitney's U-test.

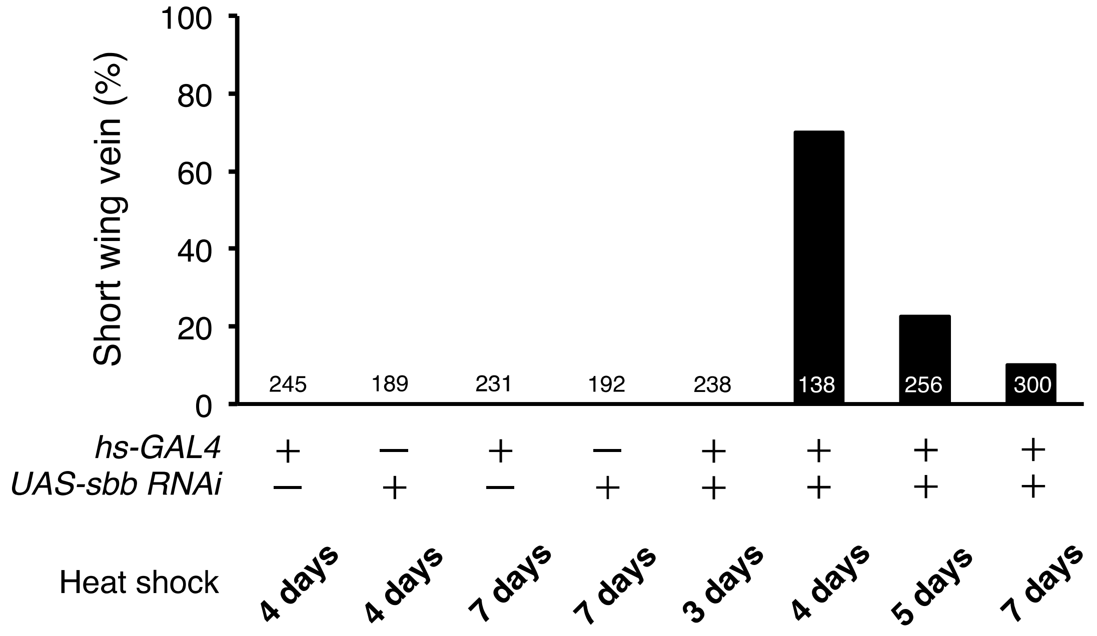

Supplementary Figure 3. The short wing vein phenotype of *plt* was reproduced by *sbb* knockdown at the larval stage. The wing vein phenotype of emerged flies carrying *hs-GAL4* and *UAS-sbb RNAi* (red bars) and that of the negative control groups. A heatshock of 37°C was applied for 1 hr at the indicated stages. The number of flies examined is indicated below each bar.

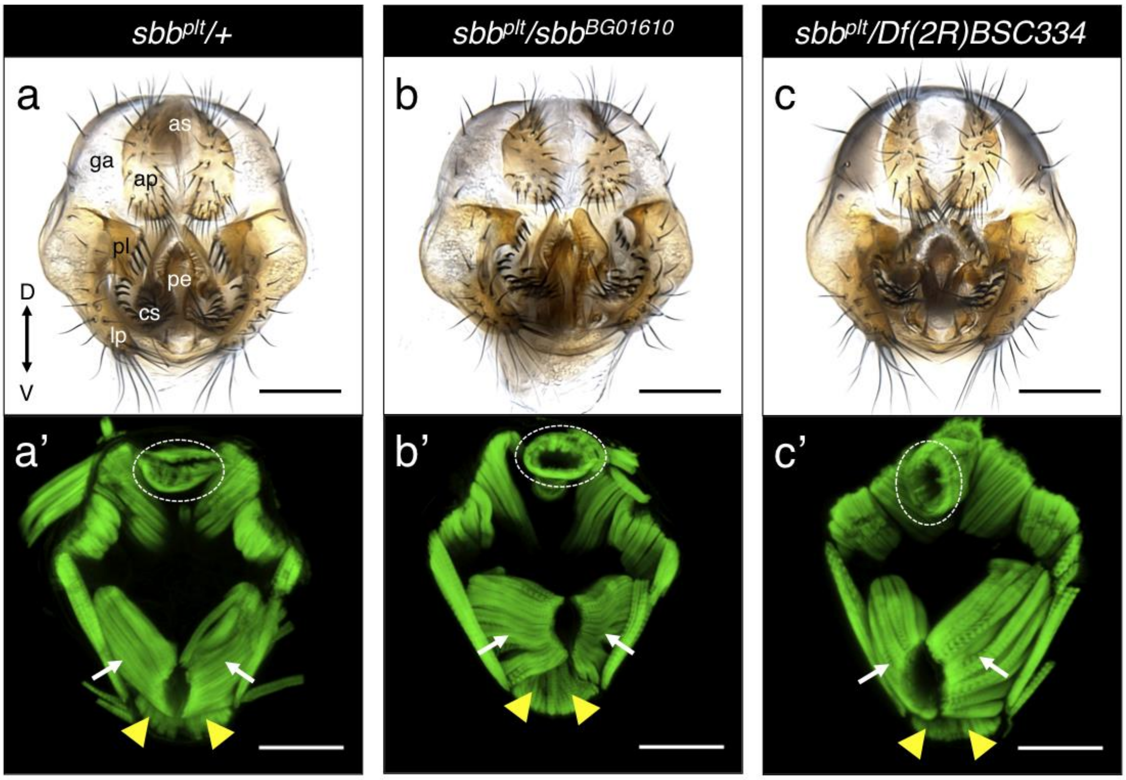

Supplementary Figure 4. Structure of genitalia and associated muscles in *sbb* heterozygous and mutant males. External genitalia (**a**-**c**) and genital muscles (**a’**-**c’**) of *sbb*^{plt}/+ (**a** and **a’**), *sbb*^{plt}*/Df(2R)BSC334* (**b** and **b’**) and *sbb*^{plt}*/sbb*^{BG01610} (**c** and **c’**) are shown. The portions of genitalia are indicated with the following abbreviations: ap:, anal plate; as, anus; cs, clasper; ga, genital arch; lp, lateral plate; pe, penis; pl, posterior lobe. White arrows and yellow arrowheads indicate the protractor and retractor muscles associated with the aedeagus, respectively. Dotted circles indicate the anus. Genital muscles were stained with FITC-labeled phalloidin. Dorsal side up. Scale bar : 100 μm.

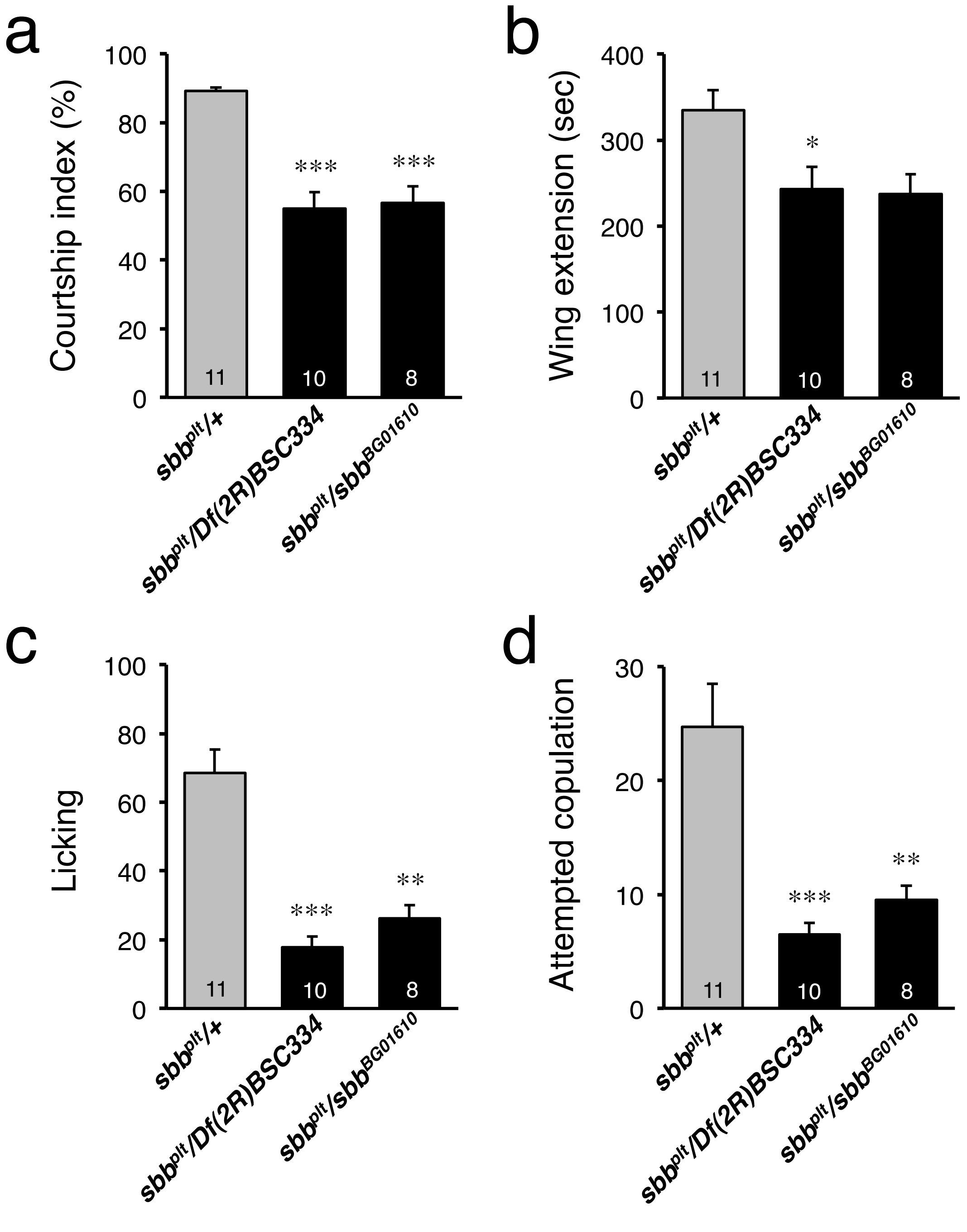

Supplementary Figure 5. Quantitative comparisons of mating activities between *sbb* mutant and control males. Shown are the courtship index (**a**), the time spent for wing extension (**b**), and the number of occurrences of licking (**c**) or attempted copulation (**d**) during a 10-min observation period. *plt* hemizygous males ($sbb^{plt}/Df(2R)BSC334$ and $sbb^{plt}/sbb^{BG01610}$) engaged in licking and attempted copulation less often than control males ($sbb^{plt}/+$) did. The graphs represent the mean ± SEM of the

indicated parameters and the numbers on the bars represent the numbers of flies examined. ***$P<0.001$, **$P<0.01$ and *$P<0.05$ by the Kruskal-Wallis test with Steel-Dwass' multiple pair-wise post hoc comparisons.

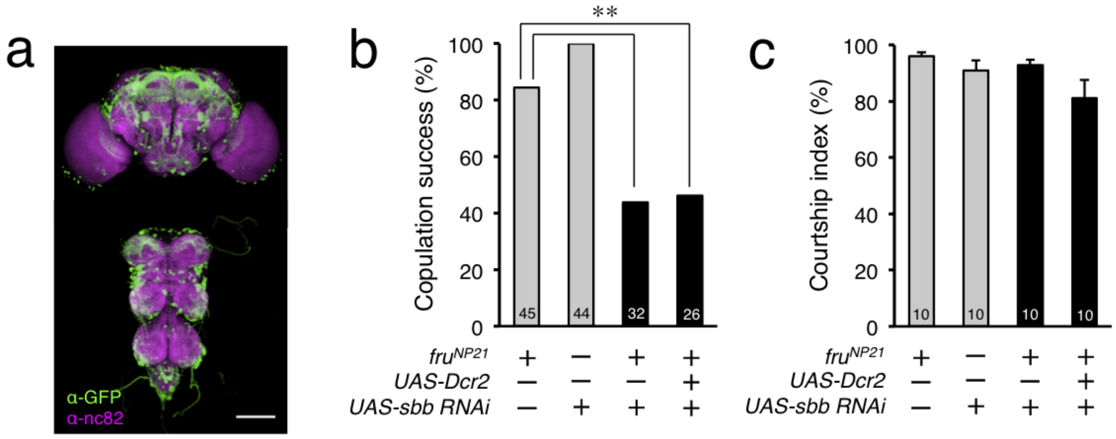

Supplementary Figure 6. *sbb* knockdown in the *fru*-expressing cells as driven by *fru*^{NP21} led a weak copulation defect. (a) Image of the CNS with *fru*^{NP21}-positive neurons. The genotype of flies is *w; UAS-mCD8::GFP/+; fru*^{NP21}/+. Scale bar: 100 µm. **(b)** The copulation success within 60 min in flies carrying *fru*^{NP21} and *UAS-sbb RNAi* in the presence and absence of *UAS-Dcr2* and that in the control flies, *fru*^{NP21}/+ and *UAS-sbb RNAi/+*. The copulation success was significantly decreased in the flies with *sbb* knockdown. (**c**) The courtship index calculated during a 10 min observation period. The number of flies examined is indicated on each bar. **P<0.01 by the Chi-square test.

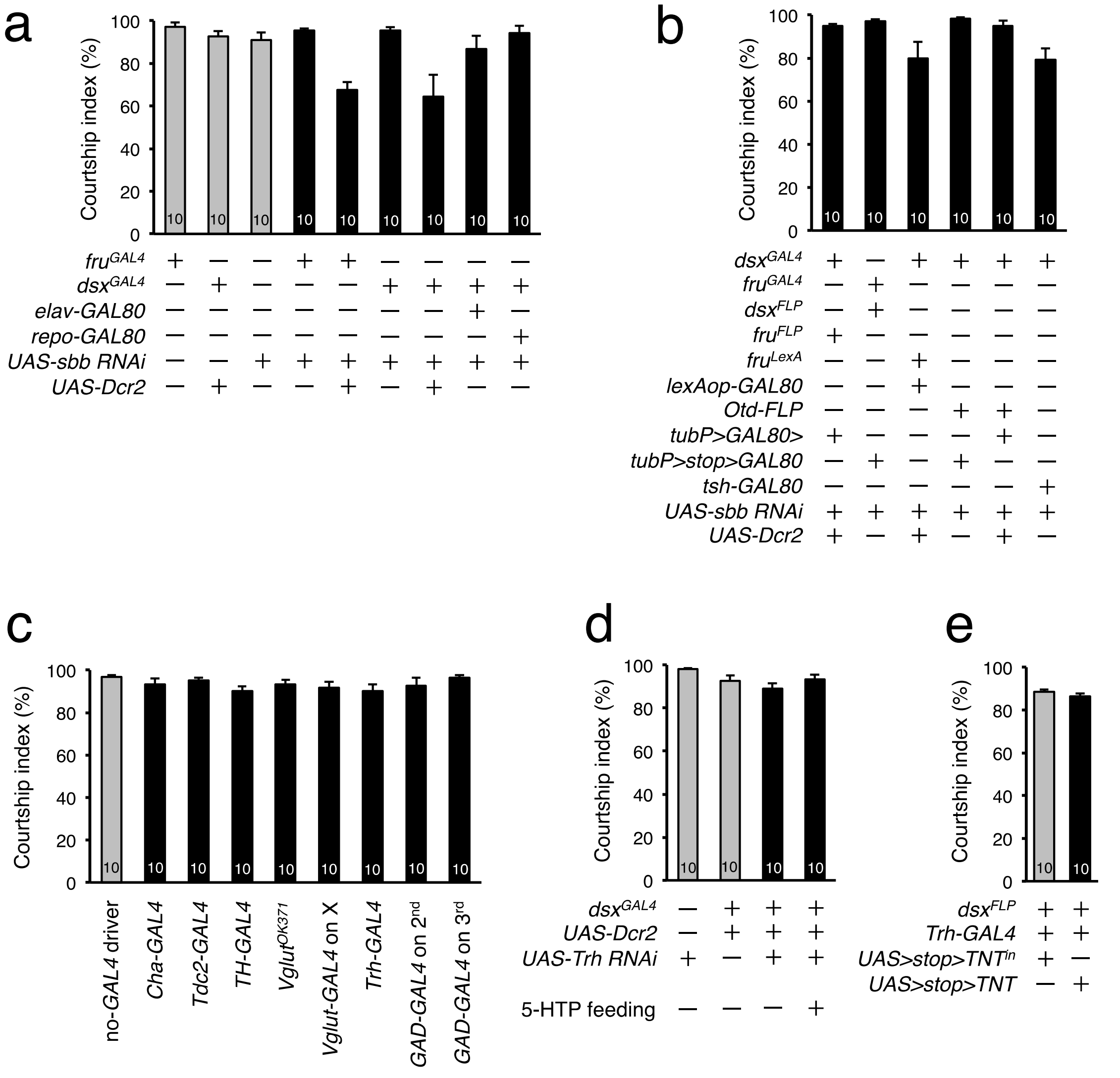

Supplementary Figure 7. Courtship vigor was barely affected in male flies with *sbb* knockdown, *Trh* knockdown or synaptic block in *dsx*[+]/*Trh*[+] cells. The courtship index of male flies in which *UAS-sbbRNAi* was expressed in different cell populations: all fru^{GAL4}-positive cells (4th and 5th bars in **a**), dsx^{GAL4}-positive cells (6th and 7th bars in **a**), dsx^{GAL4}-possitive cells other than neurons (8th bar in **a**), dsx^{GAL4}-positive cells other than glia (9th bar in **a**), dsx^{GAL4}- and fru^{GAL4}- double-positive cells (1st bar in **b**), the dsx^{GAL4}-negative subset of fru^{GAL4}-positive cells (2nd bar in **b**), the fru^{GAL4}-negative subset of dsx^{GAL4}-positive cells (3rd bar in **b**), dsx^{GAL4}-positive cells outside the head (4th bar in **b**), dsx^{GAL4}-positive cells inside the head (5th bar in **b**), dsx^{GAL4}-positive cells outside the thorax (6th bar in **b**), cells defined by different neurotransmitter-GAL4s (**c**), dsx^{GAL4}-positive cells with *Trh* knockdown in flies fed on a normal or

5-HTP-supplemented food (**d**), and *dsx*- and *Trh*-double positive cells with the expression of active (*TNT*) or inactive (TNT^{in}) tetanus toxin light chain (**e**). The copulation success rates estimated for these flies are shown in Figures 2c-g, 3a-d and 4d-f.

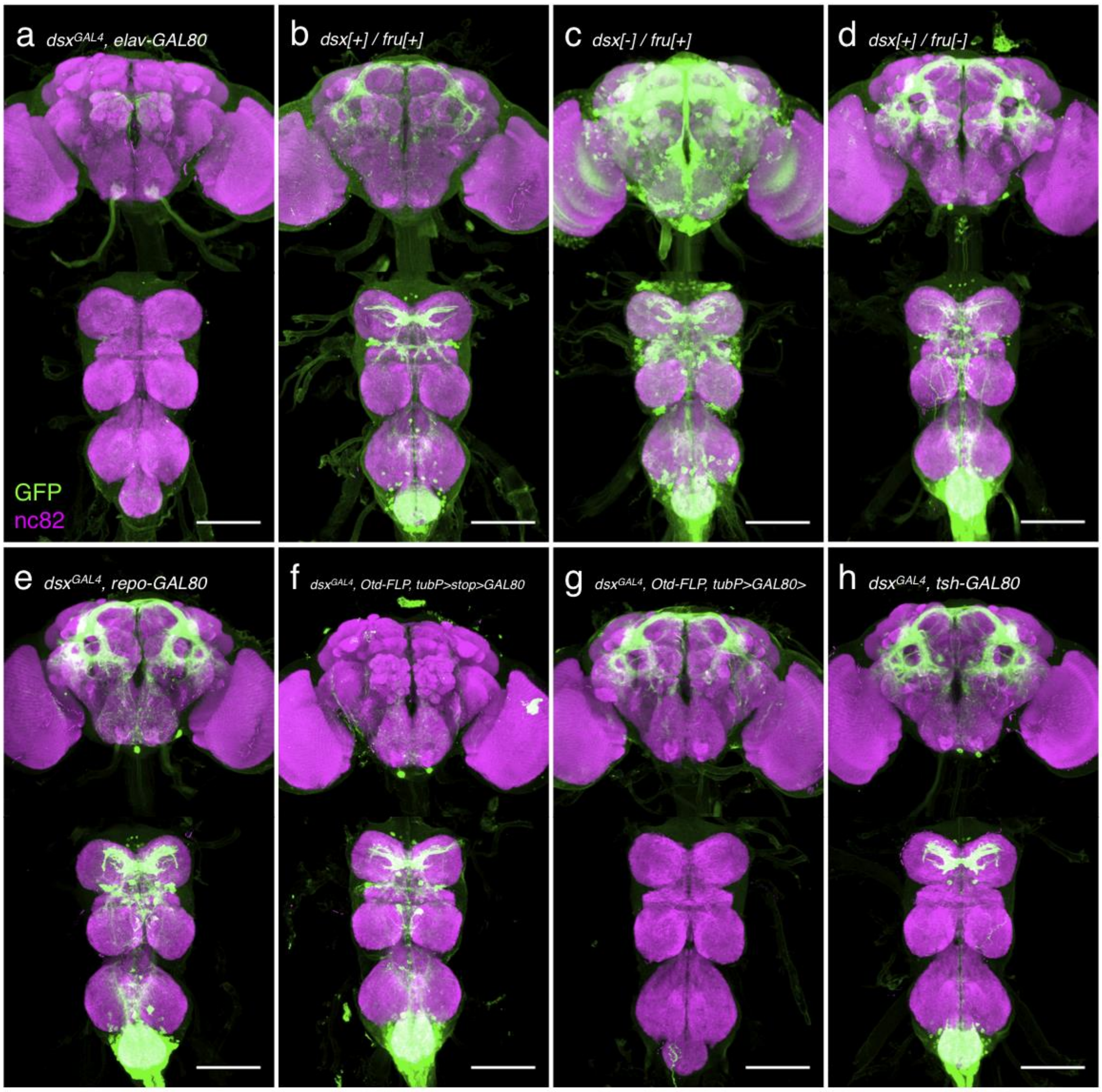

Supplementary Figure 8. Expression of GAL4 in flies tested for the effects on copulation success of *sbb* knockdown. GAL4 expression was detected in: *dsx*^{GAL4}-positive cells other than neurons (**a**), *dsx*^{GAL4}- and *fru*^{GAL4}- double positive cells (**b**), the *dsx*^{GAL4}-negative subset of *fru*^{GAL4}-positive cells (**c**), the *fru*^{GAL4}-negative subset of *dsx*^{GAL4}-positive cells (**d**), *dsx*^{GAL4}-positive cells other than glia (**e**), *dsx*^{GAL4}-positive cells outside the head (**f**), *dsx*^{GAL4}-positive cells inside the head (**g**), and *dsx*^{GAL4}-positive cells outside the thorax (**h**). Scale bar: 100 μm. GAL4 expression was detected by a GFP reporter (green) in the brain which was counterstained with nc82 (magenta). The copulation success rates estimated for these flies are shown in Figures 2c-g and 3a-c.

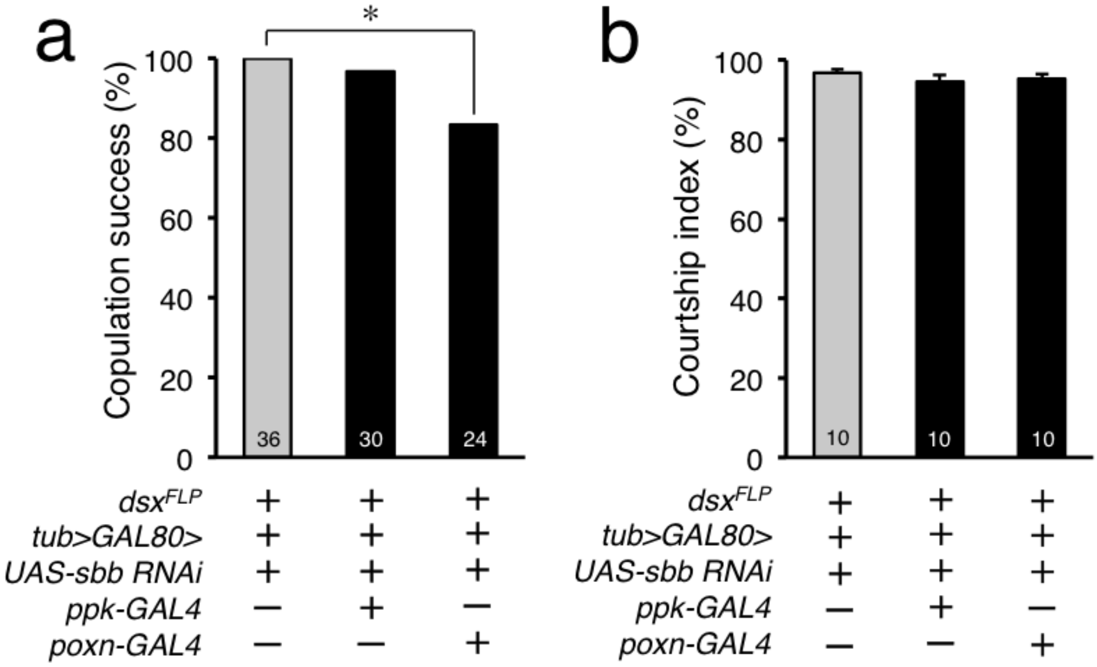

Supplementary Figure 9. *sbb* knockdown in *dsx*$^{+}$/*ppk*$^{+}$ or *dsx*$^{+}$/*poxn*$^{+}$ neurons. (**a**) The mating success of flies with the genotype of *dsx*^{FLP}, *UAS-sbb RNAi, tubP>GAL80>* with *ppk-GAL4* or *poxn-GAL4* and of the control flies, *dsx*^{FLP}, *UAS-sbb RNAi, tubP>GAL80>* without *GAL4* drivers. (**b**) The courtship index calculated during a 10 min observation period for the flies with indicated genotypes. The number of flies examined is indicated on each bar. *P<0.05 by the Chi-square test.

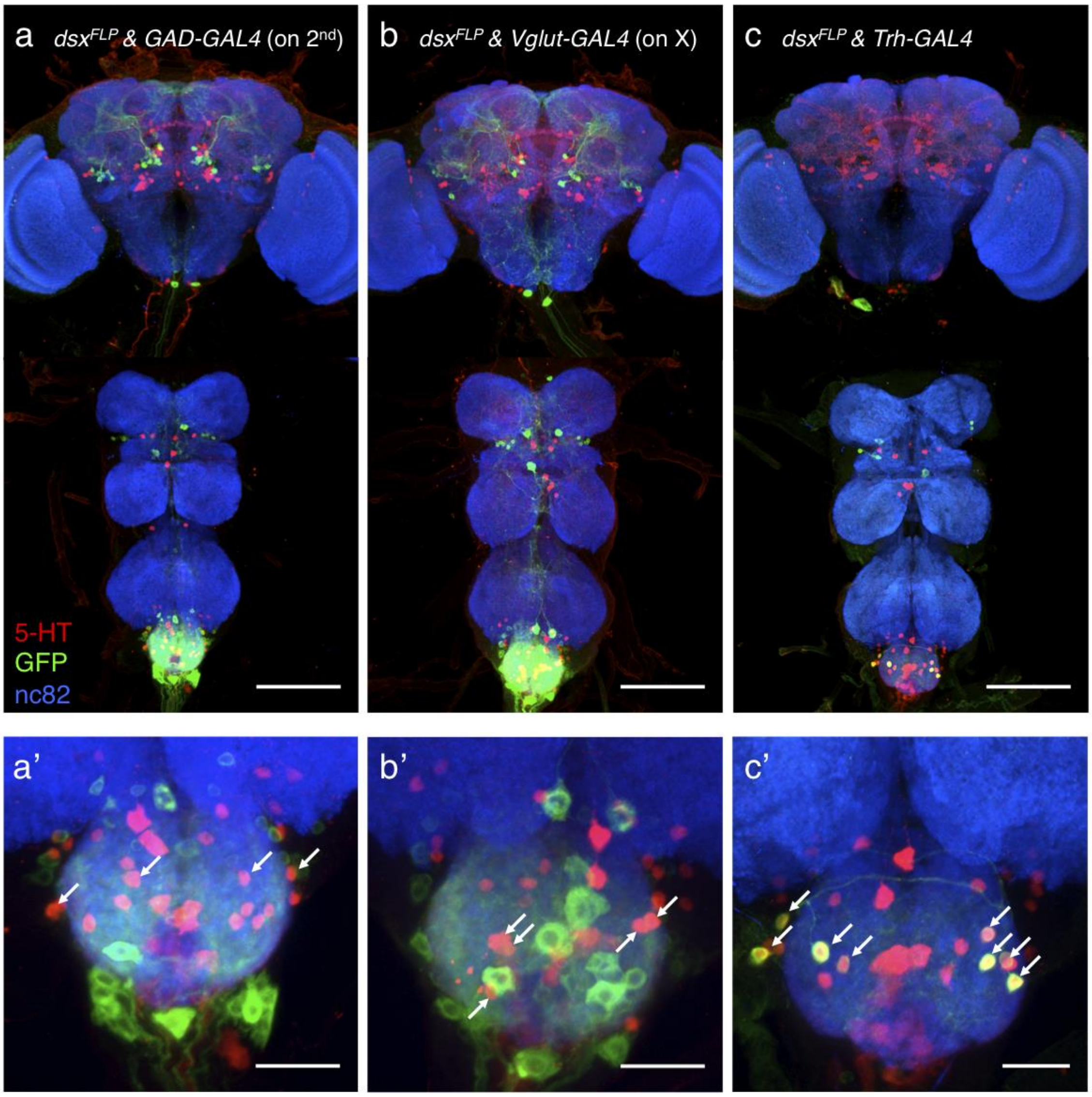

Supplementary Figure 10. 5-HT and GAL4 expression in flies used for the identification of CNS cells that affect copulation success. Anti-5-HT immunoreactivity of *dsx*-expressing cells that are also positive for *GAD-GAL4* (**a** and **a'**), *Vglut-GAL4* (**b** and **b'**) or *Trh-GAL4* (**c** and **c'**). The abdominal ganglia are shown with a higher magnification in (**a'**) –(**c'**). GAL4 expression was monitored with a GFP reporter (green) in the CNS that was also stained with the anti-5-HT antibody (red) and nc82 (magenta). Some of the cells doubly positive for 5-HT and GFP in the abdominal ganglia are indicated with arrows in (**a'**) - (**c'**). The genotypes of flies are *w; GAD-GAL4/UAS>stop>mCD8::GFP; dsx*^{FLP}/+ (**a**), *Vglut-GAL4* on X*; UAS>stop>mCD8::GFP/+ ; dsx*^{FLP}/+ (**b**) and *w; Trh-GAL4/UAS>stop>mCD8::GFP; dsx*^{FLP}/+ (**c**), respectively. Scale bar : 100 μm (**a**, **b** and **c**) and 20 μm (**a'**, **b'** and **c'**).

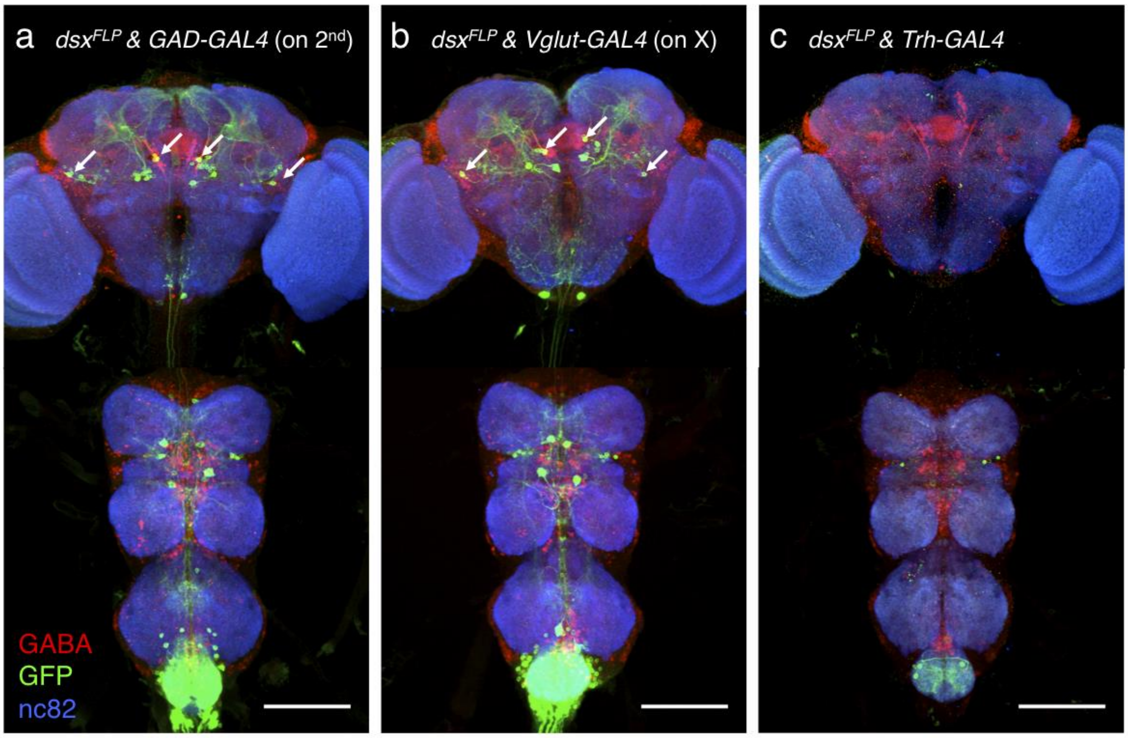

Supplementary Figure 11. GABA and GAL4 expression in flies used for the identification of CNS cells that affect copulation success. Anti-GABA immunoreactivity of *dsx*-expressing cells that are also positive for *GAD-GAL4* (**a**), *Vglut-GAL4* (**b**) or *Trh-GAL4* (**c**). GAL4 expression was monitored with a GFP reporter (green) in the CNS that was also stained with the anti-GABA antibody (red) and nc82 (magenta). Some of the cells doubly positive for GABA and GFP in the brain are indicated with arrows in (**a**) and (**b**). Scale bar: 100 μm.

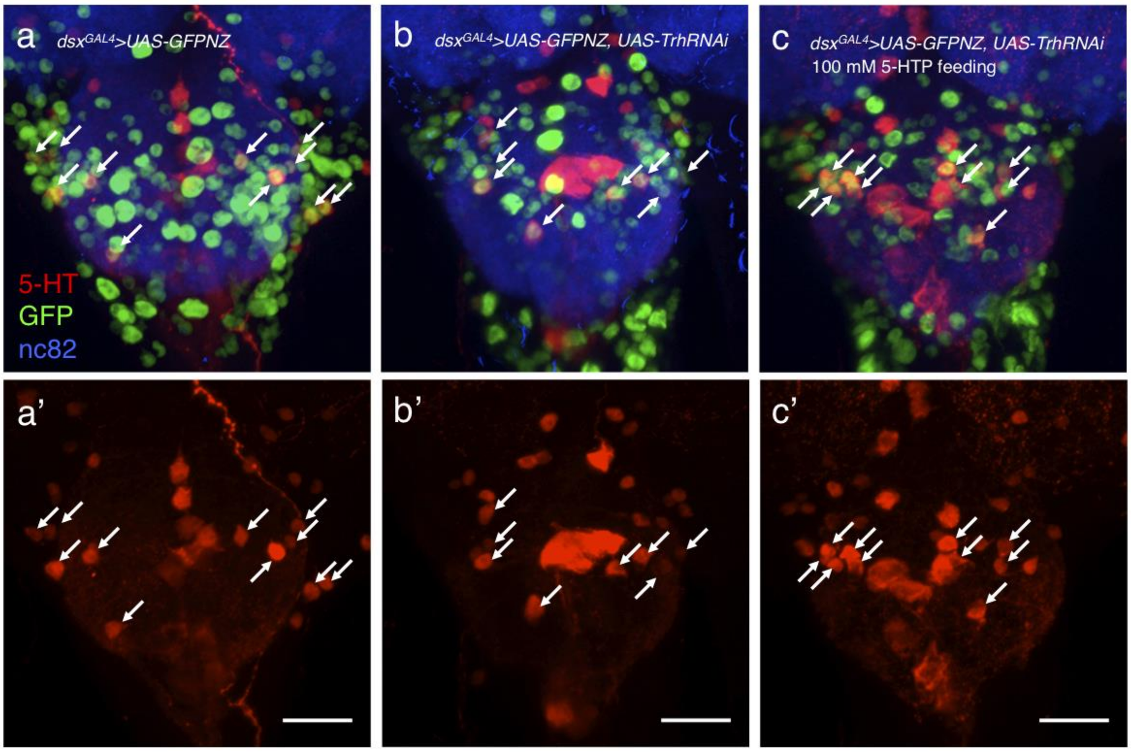

Supplementary Figure 12. Effects of *Trh* knockdown and subsequent feeding of 5-HTP on neuronal 5-HT expression. Cells in the abdominal ganglia immunoreactive to the anti-5-HT antibody (red) in a control fly (**a** and **a'**) and flies in which *Trh* was knocked down fed on a diet without (**b** and **b'**) or with (**c** and **c'**) 100 mM 5-HTP after eclosion. The genotypes of flies were *w; UAS-GFPNZ/+; dsx*^{GAL4}*/+* (**a**) and *w; UAS-GFPNZ/UAS-Dcr2; dsx*^{GAL4}*/UAS-TrhRNAi* (**b** and **c**). Scale bar: 20 μm. *dsx*-expressing cells were labeled with nuclear localizing GFP (green). The cells double-positive for *dsx* and 5-HT (*dsx*[+]/5-HT[+]) are indicated with arrows. The mean ± SEM of the number of *dsx*[+]/5-HT[+] cells was 10 ± 0.3 (n=6) in control flies, 7.7 ± 0.3 (n=7) in flies with *Trh* knockdown fed on a normal diet, and 11.5 ± 0.7 (n=4) in flies with *Trh* knockdown fed on a 5-HTP supplemented diet. The value for the flies with *Trh* knockdown fed on a normal diet was significantly smaller than that for control and for 5-HTP-fed flies with *Trh* knockdown, whereas the values for the latter two groups were statistically indistinguishable from each other ($P<0.05$ by the Kruskal-Wallis test with Steel-Dwass' multiple pair-wise post hoc comparisons).

Supplementary Note

Detailed genotypes of all strains used in the paper as follows:

Figure 1a, Supplementary Figure 1b (from left to right)

plt/+

plt/Df(2R)BSC334

plt/Df(2R)ED3683

plt/Df(2R)BSC483

plt/Df(2R)Exel7153

plt/Df(2R)BSC335

plt/Df(2R)BSC399

plt/sbb^{BG01610}

plt/sbb^{6}

plt/sbb^{k00702}

Figure 1d, Supplementary Figure 3

w; UAS-sbbRNAi/+; hs-GAL4/+

Figure 2a (left)

w; UAS-mCD8::GFP/+; fru^{GAL4}*/+*

Figure 2a (right)

+;; fru^{GAL4}*/+*

w; UAS-sbbRNAi/+

w; UAS-sbbRNAi/+; fru^{GAL4}*/+*

w; UAS-sbbRNAi/+; fru^{GAL4}*/UAS-Dcr2*

Figure 2b (left)

w; UAS-mCD8::GFP/+; dsx^{GAL4}*/+*

Figure 2a (right)

+;; dsx^{GAL4}*/+*

w; UAS-sbbRNAi/+

w; UAS-sbbRNAi/+; dsx^{GAL4}*/+*

w; UAS-sbbRNAi/UAS-Dcr2; dsx^{GAL4}*/+*

Figure 2c

w; UAS-sbbRNAi/+; elav-GAL80/+

w; UAS-sbbRNAi/+; dsx^{GAL4}*/+*

w; UAS-sbbRNAi/+; dsx^{GAL4}*/elav-GAL80*

Figure 2d

w; UAS-sbbRNAi/+

w; UAS-sbbRNAi/+; dsx^{GAL4}*/+*

w; UAS-sbbRNAi/+; dsx^{GAL4}*/repo-GAL80*

Figure 2e

w; UAS-sbbRNAi/+; fru^{FLP}*/+*

w; UAS-sbbRNAi/UAS-Dcr2; dsx^{GAL4}*/+*

tubP>GAL80>; UAS-sbbRNAi/UAS-Dcr2; dsx^{GAL4}*/fru*^{FLP}

Figure 2f

w; UAS-sbbRNAi/+; fru^{GAL4}*/+*

w; UAS-sbbRNAi/tubP>GAL80>; fru^{GAL4}*/+*

w; UAS-sbbRNAi/tubP>GAL80>; fru^{GAL4}*/dsx*^{FLP}

Figure 2g

w; UAS-sbbRNAi/+; fru^{LexA}*, lexAop-GAL80/+*

w; UAS-sbbRNAi/UAS-Dcr2; dsx^{GAL4}*/+*

w; UAS-sbbRNAi/UAS-Dcr2; dsx^{GAL4}*/fru*^{LexA}*, lexAop-GAL80*

Figure 3a

w; Otd-FLP/+; dsx^{GAL4}*/+*

w; Otd-FLP/UAS-sbbRNAi; dsx^{GAL4}*/+*

w; Otd-FLP/UAS-sbbRNAi; dsx^{GAL4}*/UAS>stop>GAL80*

Figure 3b

w; Otd-FLP/+; dsx^{GAL4}*/+*

tubP>GAL80>; UAS-sbbRNAi/+; UAS-Dcr2/+

w; UAS-sbbRNAi/Otd-FLP; UAS-Dcr2/dsx^{GAL4}

tubP>GAL80>; UAS-sbbRNAi/Otd-FLP; UAS-Dcr2/dsx^{GAL4}

Figure 3c

w; UAS-sbbRNAi/+

w; UAS-sbbRNAi/+; dsx^{GAL4}*/+*

w; UAS-sbbRNAi/tsh-GAL80; dsx^{GAL4}*/+*

Figure 3d

w; UAS-sbbRNAi, tubP>GAL80>/+; dsx^{FLP}*/+*

w; UAS-sbbRNAi, tubP>GAL80>/Cha-GAL4, UAS-GFP; dsx^{FLP}*/+*

w; UAS-sbbRNAi, tubP>GAL80>/Tdc2-GAL4; dsx^{FLP}*/+*

w; UAS-sbbRNAi, tubP>GAL80>/+; dsx^{FLP}*/TH-GAL4*

w; UAS-sbbRNAi, tubP>GAL80>/Vglut-GAL4^{OK371}*; dsx*^{FLP}*/+*

Vglut-GAL4 on X*; UAS-sbbRNAi, tubP>GAL80>/+; dsx*^{FLP}*/+*

w; UAS-sbbRNAi, tubP>GAL80>/Trh-GAL4; dsx^{FLP}*/+*

w; UAS-sbbRNAi, tubP>GAL80>/GAD-GAL4 on 2nd*; dsx*^{FLP}*/+*

w; UAS-sbbRNAi, tubP>GAL80>/+; dsx^{FLP}*/GAD-GAL4* on 3rd

Figure 3e

w; GAD-GAL4 on 2nd*/UAS>stop>mCD8::GFP; dsx*^{FLP}*/+*

Figure 3f

w; UAS>stop>mCD8::GFP/+; dsx^{FLP}*/GAD-GAL4* on 3rd

Figure 3g

w; Trh-GAL4/UAS>stop>mCD8::GFP; dsx^{FLP}*/+*

Figure 3h

Vglut-GAL4 on X*; UAS>stop>mCD8::GFP/+ ; dsx*^{FLP}*/+*

Figure 4a

w; Trh-GAL4/UAS>stop>mCD8::GFP; dsx^{FLP}*/+*

Figure 4b

w; Trh-GAL4/UAS-sbbRNAi, tubP>GAL80>; dsx^{FLP}/ UAS>stop>mCD8::GFP

Figure 4c

w; Trh-GAL4/UAS>stop>mCD8::GFP; dsx^{FLP}/+

Figure 4d

+;; UAS-TrhRNAi/+

w; UAS-Dcr2/+; dsx^{GAL4}/+

w; UAS-Dcr2/+; dsx^{GAL4}/UAS-TrhRNAi

Figure 4e

w; UAS>stop>TNT^{in}/Trh-GAL4; dsx^{FLP}/+

w; UAS>stop>TNT/Trh-GAL4; dsx^{FLP}/+

Figure 4f

w; UAS-Dcr2/+; dsx^{GAL4}/UAS-TrhRNAi

Supplementary Figure 2a

sbb^{plt}/DF(2R)BSC334

Supplementary Figure 2b

elav^{c155}-GAL4 w ; UAS-sbbRNAi/+; UAS-Dcr2/+

Supplementary Figure 4 and 5

sbb^{plt}/+

sbb^{plt}/Df(2R)BSC334

sbb^{plt}/sbb^{BG01610}

Supplementary Figure 6a

w; UAS-mCD8::GFP/+; fru^{NP21}/+

Supplementary Figure 6b and 6c

+;; fru^{NP21}*/+*

w; UAS-sbbRNAi/+

w; UAS-sbbRNAi/+; fru^{NP21}*/+*

w; UAS-sbbRNAi/+; fru^{NP21}*/UAS-Dcr2*

Supplementary Figure 7

same genotypes used in Figures 2c-g, 3a-d and 4d-f.

Supplementary Figure 8

(a) *w; UAS-mCD8::GFP/+; dsx*^{GAL4}*/elav-GAL80*

(b) *w; UAS>stop>mCD8::GFP/+; dsx*^{GAL4}*/fru*^{FLP}

(c) *w; UAS-mCD8::GFP/tubP>stop>GAL80; fru*^{GAL4}*/dsx*^{FLP}

(d) *w; UAS-mCD8::GFP/+; dsx*^{GAL4}*/fru*^{LexA}*, lexAop-GAL80*

(e) *w; UAS-mCD8::GFP/+; dsx*^{GAL4}*/repo-GAL80*

(f) *w; UAS-mCD8::GFP/Otd-FLP; dsx*^{GAL4}*/tubP>stop>GAL80*

(g) *w; UAS-mCD8::GFP/Otd-FLP; dsx*^{GAL4}*/tubP>GAL80>*

(h) *w; UAS-mCD8::GFP/tsh-GAL80; dsx*^{GAL4}*/+*

Supplementary Figure 9

w; UAS-sbbRNAi, tub>GAL80>/+; dsx^{FLP}*/+*

ppk-GAL4; UAS-sbbRNAi, tub>GAL80>/+; dsx^{FLP}*/+*

w; UAS-sbbRNAi, tub>GAL80>/+; dsx^{FLP}*/poxn-GAL4-14-1-7*

Supplementary Figure 10 and 11

(a) *w; UAS>stop>mCD8::GFP/GAD-GAL4* on 2nd*; dsx*^{FLP}*/+*

(b) *Vglut-GAL4* on X*; UAS>stop>mCD8::GFP/+; dsx*^{FLP}*/+*

(c) *w; UAS>stop>mCD8::GFP/Trh-GAL4; dsx*^{FLP}*/+*

Supplementary Figure 12

(a) *w; UAS-GFPNZ/+; dsx*^{GAL4}*/+*

(b and c) *w; UAS-GFPNZ/UAS-Dcr2; dsx*^{GAL4}*/UAS-TrhRNAi*
